# Predicting the Next Superspreader

**DOI:** 10.1128/msystems.01199-22

**Published:** 2023-01-19

**Authors:** Alfredo Chavez-Arroyo, Andreas J. Bäumler

**Affiliations:** a Department of Medical Microbiology and Immunology, School of Medicine, University of California at Davis, Davis, California, USA

**Keywords:** Salmonella, antibiotic resistance, gut microbiome, swine

## Abstract

The spread of multidrug-resistant zoonotic pathogens, such as Salmonella, within livestock is of concern for food safety. The spread of Salmonella on the farm is escalated by superspreaders, which shed the pathogen at high numbers with their feces. However, there are currently no biomarkers to identify potential superspreaders. Kempf and coworkers determined that a potent early inflammatory response to Salmonella infection and changes in the microbiota composition are associated with the superspreader phenotype in pigs (F. Kempf, G. Cordoni, A.M. Chaussé, R. Drumo, et al., *mSystems*, in press, https://doi.org/10.1128/msystems.00852-22). Since these biomarkers only develop during Salmonella infection, additional work is needed to predict animals that have the potential to become superspreaders.

## COMMENTARY

An outbreak caused by a multidrug-resistant Salmonella enterica subsp. *enterica* serovar Typhimurium clone, known as monophasic *S.* Typhimurium, started in 2006 in the United Kingdom and other European countries and is ongoing (1). Monophasic *S.* Typhimurium carries a deletion of the *fljAB* operon and is particularly associated with intestinal carriage in pig herds, from where it was introduced into the human food supply (2–4). The emergence of monophasic *S.* Typhimurium coincided with the beginning of a European Union-wide ban of using antibiotics as growth promoters in pig feed, which made inclusion of copper salts a popular alternative to improve growth performance of pig herds (5). Whole-genome analysis of monophasic *S.* Typhimurium isolates from the United Kingdom showed that they form a single clade derived from an ancestral organism carrying a large novel genomic island (designated SGI-4), suggesting that SGI-4 was acquired shortly before clonal expansion of the monophasic *S.* Typhimurium clade (6). SGI-4 encodes a heavy metal RND-family efflux pump conferring enhanced resistance to copper sulfate, thus correlating with the common use of dietary copper supplementation in the porcine reservoir from where this clade originates (5).

The phylogenetic tree of *S.* Typhimurium branches into two major subdivisions, one containing the monophasic *S.* Typhimurium clade and the other including commonly used *S.* Typhimurium laboratory strains (e.g., ATCC 14028, SL1344) as well as multidrug-resistant *S.* Typhimurium clones associated with previous outbreaks in cattle and humans (7). Knowledge about *S.* Typhimurium pathogenesis is largely derived from studies on isolates belonging to the latter subdivision, whereas the monophasic *S.* Typhimurium clade remains poorly studied. Here, Kempf and coworkers investigated which properties are associated with high fecal shedding of monophasic *S. Typhimurium* in pigs (8).

In mice, a fraction of animals, termed superspreaders, exhibit a high luminal abundance of *S.* Typhimurium within the colon and are responsible for pathogen transmission (9). The luminal abundance of *S.* Typhimurium within the colon is controlled initially by the microbiota (10), but it increases once virulence factors trigger intestinal inflammation (11). Increased pathogen growth occurs as the host response escalates the availability of respiratory electron acceptors in the colonic lumen, from which the pathogen then benefits (12–15). *S.* Typhimurium virulence factors trigger severe acute intestinal inflammation in mice (16, 17) and cattle (18), but the pathogen causes less severe disease in pigs. Nonetheless, virulence factors enable *S.* Typhimurium to overcome colonization resistance conferred by the microbiota in this host species (19). The ability to reach superspreader status is thought to be important for transmission within pig herds, which is an important food safety concern.

The new research shows that enhanced shedding of monophasic *S.* Typhimurium with the feces of pigs is associated with higher proinflammatory cytokine levels during, but not prior to, infection (8). Furthermore, the superspreader phenotype of monophasic *S.* Typhimurium in pigs is associated with changes in the microbiota composition during infection that predict a functional enrichment for pathways involved in anaerobic respiration (8). These data are consistent with previous work suggesting that *S.* Typhimurium virulence factors trigger intestinal inflammation to increase the availability of respiratory electron acceptors that boost pathogen growth (20, 21). Nevertheless, this work highlights that ongoing work is needed to define the biomarkers that reliably predict which animals will reach superspreader status after they become infected with monophasic *S.* Typhimurium.
